# Current and Future Trends of Colorectal Cancer Treatment: Exploring Advances in Immunotherapy

**DOI:** 10.3390/cancers16111995

**Published:** 2024-05-24

**Authors:** Taxiarchis Konstantinos Nikolouzakis, Emmanuel Chrysos, Anca Oana Docea, Persefoni Fragkiadaki, John Souglakos, John Tsiaoussis, Aristidis Tsatsakis

**Affiliations:** 1 Department of General Surgery, University General Hospital of Heraklion, 71110 Heraklion, Greece; konstan10@hotmail.gr (T.K.N.); chrysose@uoc.gr (E.C.); 2 Department of Toxicology, University of Medicine and Pharmacy of Craiova, 200349 Craiova, Romania; 3 Department of Forensic Sciences and Toxicology, Faculty of Medicine, University of Crete, 71003 Heraklion, Greece; persefoni_fragkiadaki@imbb.forth.gr (P.F.); tsatsaka@uoc.gr (A.T.); 4 Laboratory of Translational Oncology, Medical School, University of Crete, 70013 Heraklion, Greece; souglak@uoc.gr; 5 Department of Anatomy, Medical School, University of Crete, 70013 Heraklion, Greece; tsiaoussis@uoc.gr

**Keywords:** colorectal cancer, immunotherapy, telomeres, therapy, checkpoint inhibitors, adoptive cell therapy

## Abstract

**Simple Summary:**

Colorectal cancer (CRC) has become one of the leading causes of cancer-related morbidity and mortality worldwide. Immune checkpoint inhibitors (ICIs) and adoptive cell therapy (ACT) have been recently added to the clinical armamentarium of stage II/III diseases. Moreover, the improvement of a patient’s telomere status may have a synergistic effect along with ICIs/ACT. This can be achieved either via lifestyle modifications with increased physical activity or with new promising treatments such as those acting on telomerase. Therefore, this review aims to comprehensively summarize the current data on immunotherapy in order to facilitate clinicians’ familiarity with this relatively new therapeutic approach.

**Abstract:**

Cancer of the colon and rectum (CRC) has been identified among the three most prevalent types of cancer and cancer-related deaths for both sexes. Even though significant progress in surgical and chemotherapeutic techniques has markedly improved disease-free and overall survival rates in contrast to those three decades ago, recent years have seen a stagnation in these improvements. This underscores the need for new therapies aiming to augment patient outcomes. A number of emerging strategies, such as immune checkpoint inhibitors (ICIs) and adoptive cell therapy (ACT), have exhibited promising outcomes not only in preclinical but also in clinical settings. Additionally, a thorough appreciation of the underlying biology has expanded the scope of research into potential therapeutic interventions. For instance, the pivotal role of altered telomere length in early CRC carcinogenesis, leading to chromosomal instability and telomere dysfunction, presents a promising avenue for future treatments. Thus, this review explores the advancements in CRC immunotherapy and telomere-targeted therapies, examining potential synergies and how these novel treatment modalities intersect to potentially enhance each other’s efficacy, paving the way for promising future therapeutic advancements.

## 1. Introduction

Recent evidence from global cancer registries places cancer of the colon and rectum (CRC) in the third place of the most common types of cancer, accounting for around 10% of all cases. Furthermore, CRC is identified as the second most common cause of cancer-related death worldwide [1]. In fact, the International Agency for Research on Cancer estimated that in 2020, more than 1.9 million new patients and 900,000 deaths of CRC occurred worldwide [2]. In the United States alone, projections for 2023 indicated approximately 153,020 new CRC diagnoses and 52,550 deaths attributed to the disease [3]. Despite a decline in the age-adjusted death rate for CRC between 1990 and 2020, the pace of this decline has decelerated over the past decade [4]. Besides epidemiological factors like global population growth, aging demographics, and the rising incidence of early-onset disease, the waning effectiveness of conventional therapeutic methods (including surgery, chemotherapy, and radiotherapy) is a significant contributing factor to this trend. Recent years have seen a surge in research focused on the comprehensive treatment of CRC, with combination therapy involving targeted agents emerging as the primary treatment approach for metastatic CRC (mCRC) patients [5,6]. However, despite ongoing advancements in medication regimens, making breakthroughs in mCRC therapy remains challenging, with prognoses still being bleak. This underscores the imperative for novel therapeutic modalities capable of either circumventing the primary cell-related mechanisms of chemotherapeutic drug resistance or enhancing the organism’s anti-tumor properties. A promising candidate that seems able to achieve, at least to some extent, this task is the immune system. In fact, solid tumors such asnon-small cell lung cancer (NSCLC) and melanoma have retrieved significant therapeutic results from immunotherapy [7,8]. Immune checkpoint inhibitors (ICIs) have allowed for persistent results in certain patients, leading to favorable results. Programmed Death-Ligand 1 (PD-L1) monoclonal antibodies used against Programmed Death 1 (PD-1) or Cytotoxic T-Lymphocyte Antigen 4 (CTLA-4) dramatically augmented the therapeutic armamentarium while also providing significant positive re-appreciation of prognosis across several solid tumors [9,10,11]. However, owing to the increased variability of the underlying immunophenotypes, it is well appreciated that the clinical benefit from this pioneering therapeutic approach can vary for different cancer types [12]. Current clinical data dictate that a portion of CRC patients with tumors where deficient mismatch repair or high microsatellite instability (dMMR/MSI-H) is identified, can benefit from the use of ICIs. This group is estimated to be around 15% of all CRC patients and 5% of those with metastatic CRC (mCRC) [13,14]. However, despite the high rate of durable responses, a fraction of them will develop immune resistance that can outweigh prolonged survival outcomes (approximately 15–29%) [12,15,16]. Therefore, it is reasonable to speculate that ICIs’ efficacy is closely related with the identification of quality tumor-specific lymphocytes in high numbers. Interestingly, adoptive cell therapy (ACT) has demonstrated promising results in cases with these characteristics [17]. ACT is a sophisticated and, more importantly, individualized anti-cancer treatment, in which blood samples are retrieved from a patient, allowing for immune cell isolation, in vitro culture and modification, and thereafter re-administration back into the patient in order to selectively tackle the cancer. Through this process, cultured cells are able to specifically identify cancer cells and induce immune responses to eliminate them [18]. In contrast to ICIs that greatly exert their action through antagonism of suppressor T-cell receptors, ACT providesits anticarcinogenic effect through isolating the expansion of a great volume of T cells that may overcome certain suppressive immunomodulatory parameters [19]. Current research in CRC epigenetics highlights the important role of telomeres and their regulating enzyme, telomerase, not only as clinical biomarkers but also as potential therapeutic targets [20,21,22,23,24,25,26,27]. However, it is also well established that immune responses may be negatively affected in cases of telomere attrition [27,28]. This is important because it is proven that in CRC patients, peripheral blood lymphocytes (PBLs) contain shorter telomeres than those of healthy individuals [29] while telomerase is also down-regulated [27]. This observation implies that improving telomere length (TL) and increasing telomerase activity (TA) may augment the efficacy of immune-mediated therapies such as ACT and ICIs [30]. In Table 1, a summary of the different therapeutic approaches in immunotherapy for CRC is presented.

## 2. Genetic Determinants of Immune Response

### 2.1. Mismatch Repair Complex and Microsatellite Status in CRC

Microsatellites (MSs) are short repeated sequences of 1–6 nucleotides located in both coding and non-coding regions scattered throughout the genome. It is observed that MSs are susceptible to duplication errors through strand slippage during DNA synthesis [31]. Despite this, the mismatch repair (MMR) system is responsible for correcting these errors [32,33]. However, a defective MMR system (dMMR) can lead to DNA mistakes and subsequent microsatellite instability (MSI). MSI is identified as an alteration of microsatellite length caused by the insertion or deletion of a repetitive unit, allowing for new microsatellite alleles to be formed [34]. Therefore, the presence of MSI indicates dMMR and hypermutability of cells [35]. According to the mutant number of microsatellite sites, MMR defects can arise from somatic mutations, epigenetic silencing (such as hypermethylation of the hMLH1 promoter in sporadic cases), or germline genetic alterations (as seen in Lynch syndrome), resulting in MSI due to point mutations in MMR genes like hMSH2, hMSH6, hMLH1, PMS2, and PMS1 [36]. The first steps of a MS profile can be evaluated using the Bethesda gene status, which includes 5 MS loci (D5S346, BAT26, D17S250, BAT25, and D2S123) [37]; however, a number of researchers have proposed an extended version of this with 10 loci. Based on this panel, MSI is categorized into three groups: MSI-low (MSI-L), where 10–30% of the loci are unstable; MSI-high (MSI-H), which exhibits ≥30% unstable loci; and microsatellite stable (MSS), with <10% unstable loci [38]. An MSI-H/dMMR phenotype is identified in roughly 15–18% of stage II CRCs, and in stage III and IV disease settings, it accounts for approximately 10% and 5% of cases, respectively [39]. However, the prognostic significance of MSI/dMMR in such stages is debated [40]. CRC exhibiting a dMMR/MSI-H profile tends to be located in the right colon, while a constellation of unfavorable characteristics such as distant lymph node metastases, peritoneal cavity spread, mucus secretion, and poor differentiation accompanies it [41,42]. Nonetheless, it has been proved that this phenotype also has increased immunogenic properties inducing high immune cell infiltration, particularly from tumor-infiltrating lymphocytes [12,43]. Therefore, this molecular subset’s immune sensitivity is attributed to the high mutational burden from MMR deficiency, resulting in an augmented presentation of immunogenic neoantigens and a rich tumor microenvironment infiltrated by immune cells [12]. In contrast, tumors with a proficient MMR (pMMR) or MSS phenotype exhibit low immunogenicity [44], leading to decreased expression of checkpoint proteins and low or no T-cell tumor infiltration, explaining their intrinsic resistance to ICIs. In order to overcome this resistance, a therapeutic armamentarium consisting of ICIs and anti-cancer agents with immunomodulatory effects (e.g., anti-VEGFR or anti-EGFR) has been proposed to enhance the immunogenic profile of these lesions and make checkpoint blockade more effective [45,46].

### 2.2. BRAFV600E

BRAF is a serine-threonine protein kinase positioned downstream in the KRAS signaling cascade. Assessing BRAFV600E mutations holds crucial significance for both effective genetic/oncologic counseling and prognostication. The coexistence of the BRAFV600E mutation with deficient mismatch repair (dMMR) strongly suggests a sporadic tumor, obviating the need for oncogenetic referral [47]. Additionally, identifying this mutation indicates a poorer outcome in both stage II and III CRC cases [48] Specifically, the presence of the BRAFV600E mutation predicts an unfavorable prognosis in right-sided microsatellite stable (MSS) CRC and undervalued effects of systemic treatment regardless of the use of targeted therapy [49]. The BRAFV600E mutation is frequently linked with the hypermethylator phenotype (CpG island methylator phenotype), which is able to induce MSI-H status via hypermethylation of the MLH1 gene promoter [50]. Recent research of four mCRC studies revealed that 21% of BRAFV600E(+) cases were also MSI-H/dMMR, while 35% of MSI-H/dMMR cases were BRAFV600E(+) [40]. Despite growing evidence indicating that dMMR/BRAFV600E(−) tumors exhibit improved overall survival (OS) compared to dMMR/BRAFV600E(+) and proficient MMR (pMMR)/BRAFV600E(−) or pMMR/BRAFV600E(+) tumors [49,51], the presence of the BRAFV600E mutation does not predict outcomes for patients who have MSI-H mCRC that is managed with ICIs. Furthermore, the KEYNOTE-177 phase III study showed pembrolizumab’s increased efficiency over first-line systemic treatment with or without additional targeted therapy, irrespective of BRAF mutational status [52]. Additionally, the efficacy of ICIs in phase II studies with dMMR/MSI and BRAFV600E tumors versus dMMR/MSI and BRAF wild type was similar [53,54,55,56].

## 3. Immune Response in CRC

**Figure 1:**
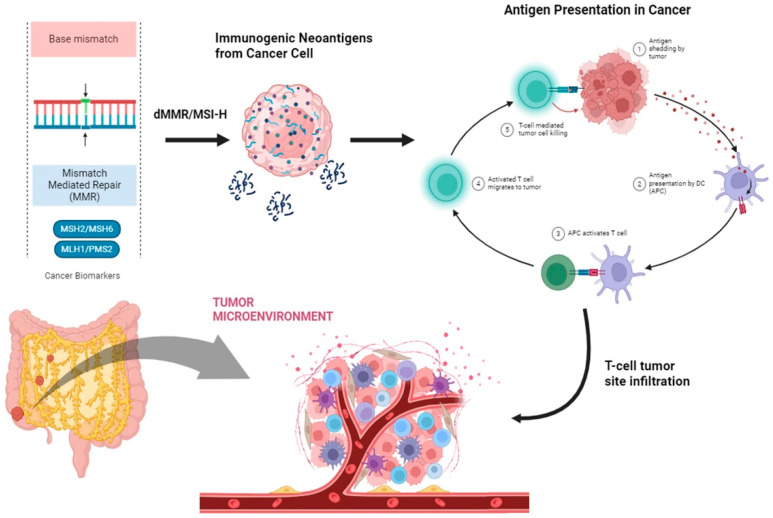
A graphical presentation of T cells infiltrating a tumor site in MSI-H CRC as a result of the increased mutational burden induced by the deficient mismatch repair system.

One of the primary mechanisms by which an organism identifies and eliminates cells that may have evaded cell-cycle regulation and become neoplastic is through its immune system. Tumor cells can activate a host’s immune system via a complex pathway encompassing not only the adaptive immune system (T and B lymphocytes) but also the innate system (such as mast cells, dendritic cells (DCs), macrophages, natural killer (NK) cells, neutrophils, and myeloid-derived suppressor cells) [57]. For an immune cell to recognize a neoplastic cell, the latter must express modified molecules on its surface, typically proteins resulting from DNA mutations or epigenetic alterations [58,59]. In a typical scenario, innate immune cells like NK cells identify the absence of MHC-I surface molecules on cancer cells, leading to the active elimination of cancer cells and subsequent recruitment of other inflammatory cells through cytokine production [60]. Monocytes recruited to the site, particularly DCs and macrophages, destroy tumor cells and transport tumor-associated antigens on their surface, initiating a specific cytolytic T-cell response targeted against the tumor [61]. This sequence of events is referred to as the cancer–immune cycle [62]. A series of studies have shown that the identification of tumor-specific T cells is linked to favorable outcomes in various human cancers [63]. Nonetheless, anti-tumor immunity is not always elucidated, as T cells can also lead to cancer progression [64]. CD8+ T cells and CD4+ effector T cells have demonstrated anti-cancer potential, while regulatory T cells (CD4+ CD25+ Tregs) have been shown to be able to contribute to the attenuated immune response exhibited in cancer [65,66]. CD8+ T cells are regarded as one of the most promising T-cell subsets for an effective anti-tumor response [65,67]. In the case of dMMR/MSI-H CRCs, there is heightened immunogenicity, leading to a significantly increased number of tumor-infiltrating CD8+ T lymphocytes (TILs) and a good prognosis [68,69]. Several immune-triggering antigens, such as mutated Caspase-5, TGFβIIR, or CDX2 have been identified in MSI-H CRCs [70]. Moreover, MSI-H results in the upregulation of immune checkpoint proteins (like PD-1 and PD-L1), facilitating immune evasion via tumor-infiltrating lymphocytes, rather than tumor cells [71]. Given the prognostic importance of TILs in the tumor microenvironment, special scores evaluating specific immunologic parameters (such as Immunoscore^®^) have been developed [72]. In CRC, the “hot” immune phenotype, characterized by significant T-cell infiltration, is often observed in tumors with MSI-H, likely owing to a high tumor mutational load and the presentation of neoantigens, fostering anti-cancer immunity (Figure 1).

## 4. Immune Evasion of CRC

Neoplastic cells must deactivate certain inhibitory mechanisms and establish new defensive strategies to ensure their survival and proliferation. Immune evasion stands out as a crucial mechanism among these strategies. Initially, immune cells work to eliminate all cancerous cells upon detection. However, some groups of tumor cells manage to surpass the immune system’s regulatory functions, achieving a balance between elimination and proliferation. Over time, a subset of cancer cells emerges that is able to evade the immune system’s suppressive effects, resulting in the creation of clinically significant cancers. This process is encapsulated by the “three Es of cancer immunoediting”: equilibrium, escape, and elimination [73]. Neoplastic cells employ various tactics to achieve immune escape, including altering surface molecules (such as the depletion or down-regulation of HLA I antigens), activating suppressor T cells, manipulating death receptor signaling responses, secreting immunosuppressive cytokines, and inducing apoptosis of activated T cells [67]. Cell surface molecules act by facilitating interactions between cells and their environment, mediating signaling pathways, and enabling cell attachment to the extracellular matrix (ECM). These molecules undergo dynamic modifications in response to intrinsic and extrinsic stimuli and vary between cell types and developmental stages. Notably, alterations in cell surface molecules contribute significantly to pathological conditions, including cancer and its metastasis [74,75]. The down-regulation of the HLA class I antigen in CRC, for instance, enables neoplastic cells to evade cytotoxic T cell lymphocyte (CTL) mediated lysis by bypassing the MHC-restricted, antigen-specific triggering of the T-cell receptor (TCR) complex [76]. Additionally, evidence supports the involvement of Fas/Fas Ligand (FasL) interplay in immune evasion [77], with neoplasms showing resistance to Fas-mediated cytotoxicity and FasL expression potentially resulting in immune effector cells apoptosis [76,78]. Furthermore, regulatory T cells (Treg), a subset of CD4+ T cells, accumulate in the tumor microenvironment, contributing to immune escape mechanisms [79]. Cancer cells also directly inhibit the immune system through molecules like vascular endothelial growth factor (VEGF), prostaglandin E2, transforming growth factor-β (TGF- β), and interleukin-10 (IL-10), which suppress cytotoxic T-cell function [77,78,80,81,82,83]. This allows for the emergence of suppressed CD 4+ and CD 8+ T lymphocytes that are not identified as foreign antigens [84]. Additionally, cancer cells can increase immune checkpoint molecules such as PD-L1, resulting in peripheral T cell exhaustion and the inhibition of malignant cell apoptosis [85].

## 5. Immune Checkpoints

### 5.1. Programmed Death Ligand 1 (PD L1) and Programmed Death 1 (PD-1)

T cell receptors Cytotoxic T lymphocyte antigen 4 (CTLA-4) and Programmed Cell Death 1 (PD-1) have a synergistic inhibitory effect on the T cell immune response. Interestingly, tumor cells take advantage of this inhibitory effect in order to induce T-cell tolerance and exhaustion [86]. A neoplasm’s microenvironment is where the PD-1/PD-L1 pathway closely controls the creation and long-term establishment of immune tolerance. It has been shown that PD-L1 or PD-L2, acting through their ligation to PD-1, may induce anti-cancer immune responses. This is achieved through the direct modulation of T-cell activity, proliferative capacity, and cytotoxicity in cancer, leading to deprived responses [87]. PD-1, also known as CD279, was initially discovered in 2B4-11 (murine T-cell hybridoma) and interleukin-3 (IL-3)-deprived LyD9 (murine hematopoietic progenitor) cell lines in 1992 [88]. Studies have demonstrated that PD-1 shares approximately 13%, 15%, and 20% similarity with induced T-cell co-stimulators CD28 and CTLA4, accordingly [89]. Functionally, PD-1 is able to exert its inhibitory effects on both innate and adaptive immune responses and is able to activate macrophages, T cells, B lymphocytes, DCs, NK, and monocytes [90]. The transcription of PD-1 is induced by various transcription factors, among which are NOTCH, interferon (IFN), regulatory factor 9 (IRF9), nuclear factor of activated T cells (NFAT), and Forkhead box protein (FOX) O1 [91]. Two well-maintained upstream regulatory regions, B and C (CR-B and COR-C), have been identified as crucial for the expression of the PD-1 gene [87]. Specifically, c-FOS connects to special areas in the CR-B region, enhancing PD-1 expression and stimulating T-cell receptors following antigen detection from naive T cells [92]. However, it is well established that neoplastic cells exploit the regulatory action of the c-FOS subunit of Activator protein 1 (AP1) by increasing its expression, thereby upregulating PD-1 expression [93]. Overall, PD-1 appears to play two opposing roles as an immune supervisor by restricting immune cells from prolonged ineffective or harmful responses, while on the other hand allowing for immune cells to develop tolerance. Another interesting way that PD-1 promotes survival and proliferation of cancer cells is via negative interference with the immune response [94]. The PD-1 ligand (PD-L1), also known as CD279 and B7-H1, is a 33kDa type 1 transmembrane glycoprotein belonging to the B7 series. It has an extracellular region with two domains (Ig- and IgC) consisting of 290 amino acids [95]. PD-L1 is typically found on the surface of dendritic cells (DCs), macrophages, and some activated T cells and B cells. In addition, if inflammation is present, PD-L1 can be expressed on certain epithelial cells [96]. Remarkably, cancer cells have managed to develop a certain “adaptive immune mechanism” by expressing PD-L1 on their surface. If so, tumor cells can evade anti-cancer immune responses [97]. A series of studies have demonstrated that PD-L1 can be upregulated by IFN-γ in various cancer models, primarily through the MEK/ERK and MYD88/TRAF6 pathways [98]. Recent studies indicate that PD-L1 upregulation on tumor cells can be achieved via IFN-γ secretion from NK cells. This is possible because of the JAK1/JAK2 and STAT1 pathways, leading to tumor cell survival and disease progression [99,100]. Indeed, tumor proliferation and survival can be mediated through the connection of PD-L1 to its receptors [101]. The PD-1/PD-L1 axis can interact with various pathways, including MAPK, PI3K/AKT, WNT, JAK/STAT, Hedgehog (Hh), and NF-κB, ultimately contributing to tumor progression [102,103,104,105,106,107,108]. Overall, PD-L1 expression has been linked to poor clinical outcomes, being more common in mCRC [109]. Interestingly, current data suggest that cancers with MSI-H exhibit upregulation of immune checkpoint proteins like PD-1 and PD-L1, facilitating immune evasion through tumor-infiltrating lymphocytes rather than tumor cells themselves [71]. Nevertheless, the expression of PD-L1 in mCRC is not associated with an immunotherapy response [110].

### 5.2. Cytotoxic T-Lymphocyte Antigen-4 (CTLA-4)

Cytotoxic T lymphocyte antigen-4 (CTLA-4) is a pivotal inhibitory receptor that modulates T-cell function and plays a crucial role in the initial phase of immune responses [111]. Also known as CD152, CTLA-4 is a 25 kDa molecule consisting of a leader peptide, a ligand binding domain, a transmembrane domain, and a cytoplasmic domain. In humans, three isoforms are produced through gene splicing: full-length CTLA-4, soluble CTLA-4, and forms involving exon 1 and exon 4 [112]. CTLA-4 exerts its inhibitory effects by influencing T-cell function, particularly during the priming phase of immune responses [113]. Its expression is upregulated in response to stronger stimulatory signals through the T-cell receptor (TCR), leading to translocation to the T cell surface [114]. On the T-cell membrane, CTLA-4 exhibits higher binding avidity to B7 molecules compared to CD28, thus competing with CD28 to regulate costimulation and induce anergy [115]. Additionally, CTLA-4 is constitutively expressed on regulatory T cells (Tregs) to maintain their suppressive functions and inhibit other T cells by interacting with B7 molecules [116]. The downstream signaling of CTLA-4 involves the induction of NF-κB, AP-1, and clustering of zeta chain-associated protein kinase 70 (ZAP-70), which are key molecules in TCR signaling pathways [117]. Furthermore, the CTLA-4/CD80, CD86 axis established between immune cells and tumor cells has been implicated in attenuating anti-tumoral immune responses in the tumor microenvironment [118]. Blocking CTLA-4 with antibodies increases CD8+ lymphocytes and depletes Tregs. However, depletion of Tregs alone does not enhance anti-tumor responses; rather, a combination of enhanced effector T cell function and the inhibition of Treg activity is necessary for maximal anti-tumor activity [119]. Anti- CTLA-4antibodies have been shown to deplete intratumoral Tregs, possibly through antibody-dependent cell-mediated cytotoxicity, leading to enhanced anti-tumor responses [120]. Additionally, studies by Allison et al. using murine colon carcinoma and fibrosarcoma models demonstrated that co-administration of anti-CTLA-4 antibodies with tumor lysate-loaded dendritic cells can reduce Tregs, increase CD8+ lymphocytes, inhibit metastatic growth, and prolong survival [121].

## 6. Immune Checkpoint Inhibitors (ICIs)

ICIs such as anti-CTLA-4, anti-PD-1, and anti-PD-L1 can bind to these co-inhibitory receptors, effectively reviving the anti-cancer immune response [122]. The US Food and Drug Administration (FDA) has approved three distinct categories of ICIs for treating various cancer types: CTLA-4 inhibitors (Ipilimumab), PD-L1 inhibitors (such as Atezolimumab, Avelumab, and Durvalumab), and PD-1 inhibitors (including Pembrolizumab, Nivolumab, and Cemiplimab).

### 6.1. Anti-PD1/PDL-1 Abs

Monoclonal antibodies targeting PD-1/PD-L1 interactions have been demonstrated to enhance T cell activity against cancer cells by restoring their activation, proliferation, function, and downstream immune signaling [123]. Among the FDA-approved PD-1 inhibitors, three are specifically approved for CRC treatment. Nivolumab, an anti-PD1 monoclonal antibody (moAb), has been utilized since 1 August 2017, for MSI-H/dMMR mCRC, as evidenced by the CheckMate 142 study [54]. Pembrolizumab, another anti-PD1 moAb, received approval on 29 June 2020, as a first-line treatment for patients with unresectable or metastatic MSI-H/dMMR CRC based on the KEYNOTE-177 study [124]. Atezolizumab, an anti-PDL1 moAb, has been investigated for previously treated mCRC with or without cobimetinib, compared to regorafenib. However, it did not achieve its primary endpoint of improved OS [125]. Nevertheless, a phase 2 study (NCT05118724) is currently ongoing, assessing the efficacy of atezolizumab with or without IMM-101 in patients with MSI-H/dMMR stage III CRC ineligible for oxaliplatin [126]. Dostarlimab, a humanized anti-PD-1 moAb, is utilized in patients with locally advanced rectal cancer that is dMMR [127].

### 6.2. Anti-CTLA-4 Abs

**Figure 2:**
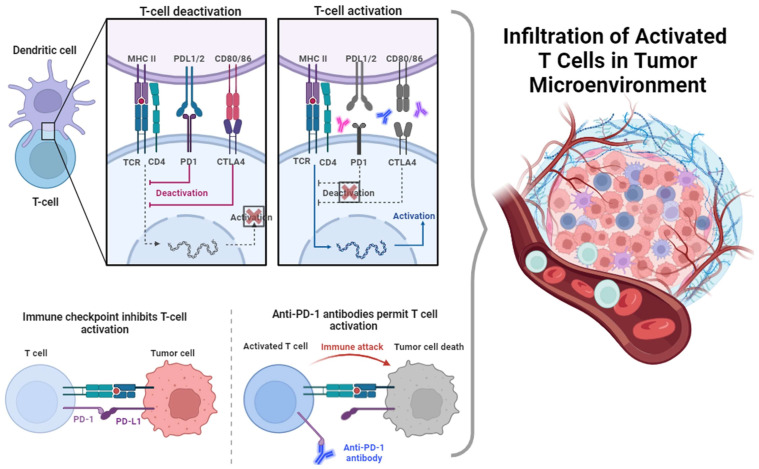
Graphical presentation of T-cell activation through the PD1/CTLA-4 pathway. Dendritic cells are able to activate T cells through the cross talk of the transmembrane proteins MHC II and TCR/CD4. However, this activating interaction can be canceled downstream if PD-L1/2 and CD80/86 expressed on the surface of dendritic cells connect with PD-1 and CTLA-4, respectively. This deactivating action can be counteracted through the inhibition of anti-PD-1/antiCTLA-4 antibodies. This allows activated T cells to enter the tumor microenvironment and eliminate cancer cells.

Two human monoclonal antibodies targeting CTLA-4 have been developed for patient use: MDX-010, also known as ipilimumab and marketed as Yervoy, and CP-675, also known as tremelimumab [128,129]. It has been shown that in a clinical setting, the use of these inhibiting anti-CTLA-4 antibodies is able to enhance the activity of effector T cells while suppressing regulatory T cells, resulting in net inhibition of tumor progression [130]. Recently, a combination of nivolumab and ipilimumab, a CTLA-4 inhibitor, gained approval from the FDA for the treatment of previously treated MSI-H/dMMR mCRC [54,131]. Figure 2 provides an illustration of the PD1/CTLA-4 T-cell pathways.

## 7. Adoptive Cell Therapy (ACT)

ACT is a highly personalized cancer treatment approach wherein immune cells are extracted from tumor patients, expanded and modified in laboratory settings, and subsequently reintroduced into the patient’s body. This process enables the modified cells to specifically target tumor cells, triggering autologous immune responses to eradicate them [18]. However, the application of ACT in solid tumors encounters significant limitations and challenges, primarily stemming from the availability and quality of isolated immune cells. Firstly, the process involves identifying and isolating specific tumor antigens to train the immune cells effectively. These cells must survive in vitro before being reintroduced into the patient [132]. Secondly, while ACT demonstrates high efficacy, it also comes with notable toxicity risks, particularly concerning cytokine release syndrome (CRS). Lastly, there are challenges associated with delivering tumor-specific lymphocytes efficiently to the tumor site [133].

### 7.1. Chimeric Antigen Receptor (CAR) T-Cell Therapy

CAR T-cells are genetically modified to produce a synthetic T-cell receptor designed to target surface antigens located on cancer cells. These receptors, known as chimeric antigen receptors (CARs), are modified proteins consisting of four parts: an extracellular antigen-binding domain, a hinge region, a transmembrane (TM) domain, and an intracellular signaling domain [134]. The extracellular domain is able to recognize and bind to tumor antigens. A single-chain fragment variable (scFv) is its main component that is created from the light and heavy chain variable region (VL and VH accordingly) of antibodies. Between the extracellular domain (antigen-binding) and the TM domain is the hinge region (also known as the spacer region). This is created from a combination of IgG4 and CD8. CARs are connected to the cell membrane via the TM domain and the hinge region. The intracellular signaling domain (that comprises CD3ζ and various synergistic proteins) has the potential to propagate immune responses that are specific to antigens and directly influence the effectiveness of CARs in activating T cells [135]. Typically, current variants of chimeric receptors contain a single-chain variable fragment portion of the target receptor, coupled with the costimulatory intracellular domain of 4-1BB, CD28 or OX40 [136]. While CAR T-cell therapy has demonstrated promising efficacy in B-cell hematologic malignancies [137], its success in treating solid tumors is less robust, possibly due to the limited availability of targetable antigens or inefficient trafficking of CAR T cells to tumor sites [130]. Nevertheless, the clinician needs to bear in mind that despite the great effectiveness of CAR T-cell therapy, there is a considerable risk of toxicity, particularly the hazard of developing cytokine release syndrome (CRS) [138]. This risk underscores the importance of the careful selection of target antigens, as they play a crucial role in determining the effectiveness and safety profile of CAR T-cell therapy. Given that the majority of solid tumors are of epithelial origin, the identification of tumor-specific antigens (TSAs) is unusual [139]. Unfortunately, given that the antigen targets used for anti-CRC CAR T-cell therapy are often expressed in normal cells, the accurate recognition of tumor-associated antigens (TAAs) is rather difficult, resulting in a therapy with low specificity [140].

### 7.2. Tumor-Infiltrating Lymphocyte (TIL) Therapy

Tumor-infiltrating lymphocytes (TILs) are isolated from samples retrieved from tissue biopsies or surgical specimens and are located close to the tumor. Following isolation, cells are proliferated in vitro using interleukin-2 (IL-2) and then are introduced back into patients in order to produce a robust immune-mediated anti-tumor response [141]. Successful TIL therapy relies on the presence of preexisting tumor-reactive lymphocytes in patients, which can be expanded ex vivo. However, identifying these specific lymphocytes, especially in patients with tumors other than melanoma, can be challenging [141]. For gastrointestinal tumors, such as CRC, the primary hurdle in developing TIL therapy may not lie in the in vitro expansion of bulk TILs but rather in the ability to select and enrich tumor-reactive T cells [142]. Despite these challenges, TIL therapy has shown promising results. For instance, in the case of metastatic CRC (mCRC), multiple lung metastatic lesions exhibited objective regression after the infusion of HLA-C*08:02-restricted TILs targeting KRAS G12D specifically [143]. As of January 2024, there are 14 clinical trials underway to assess the feasibility and safety of TIL cells for CRC treatment. Detailed information about these trials can be found on the ClinicalTrials.gov website (NCT03935893, NCT04426669, NCT03610490, NCT02980146, NCT01174121, NCT05573035, NCT05902520, NCT05610592, NCT04622423, NCT05524012, NCT03960021, NCT03904537, NCT00019084, NCT05576077).

## 8. Anti-Tumor Vaccine Therapy

The idea behind cancer vaccination originates from acknowledging that the immune system possesses inherent modules to identify modified self-antigens found on many cancer cells, commonly referred to as tumor-associated antigens [144]. As for the vaccines created for infectious diseases, the primary objective of anti-cancer vaccines is to promote an immune response aiming to eradicate tumors and establish continuous monitoring to prevent their recurrence. Up until now, Up until now, four main platforms of anti-tumor vaccines have been studied: those utilizing dendritic cells (DCs), peptide antigens, viral or bacterial vectors and those utilizing ehole tumor antigens [145]. A summary of the main immune-mediated anti-tumor vaccines is presented in Table 2.

Whole tumor vaccines: This was the first type of anti-tumor vaccine that was tested. For the majority of cases, the method involved the use of a sample of neoplastic tissue, which was submitted either to lysis or irradiation. Subsequently, it was combined with an immune adjuvant, such as alum, before being reintroduced to the donor (patient) [146]. In fact, the idea of autologous whole tumors being used as a vaccine platform has been utilized in various types of immunogenic cancers like CRC [147], renal cell carcinoma [148], and melanoma [149]. Unfortunately, despite the initial enthusiasm for whole tumor vaccines, current evidence suggests limited efficacy. For instance, in CRC, a phase III randomized clinical trial was conducted, where patients with stage II and III CRC were administered autologous whole cancer cells that were combined with a BCG vaccine. This trial aimed to assess whether surgical resection coupled with vaccination was superior to resection. However, the study failed to demonstrate a significant benefit in terms of OS or disease-free survival (DFS). Nonetheless, it was observed that both improved DFS and OS were achieved given the potent immune response [147]. An inherent challenge with whole tumor vaccines lies in the fact that only a small fraction of the proteins within an autologous whole tumor vaccine are exclusive to malignant cells. The majority of antigens contained in the vaccine are common with normal cells, which diminishes the concentration of tumor-specific antigens while potentially triggering an autoimmune response. Additionally, whole tumor vaccines tend to exhibit poor immunogenicity. Consequently, the immune response elicited by these vaccines is often inadequate to confer significant benefits to patients, as demonstrated by the average outcomes observed in previous studies [150].

Peptide antigen vaccines: Given that cancer cells express a great amount of common proteins with normal cells, this restricts immune cells from targeting them, making whole tumor vaccines less effective. On the contrary, peptide vaccines are suggested to provoke an immune reaction against a particular identified tumor antigen. These vaccines consist of entire proteins or protein fragments derived from tumor-specific proteins, administered alongside an adjuvant [151]. The main drawback is the human leukocyte antigen (HLA)-type restricted nature of the therapy. This is because HLA molecules are expressed in a variety of super-types and only a particular HLA-super-type can recognize a specific antigen. In the clinical setting, this means that only those patients who carry the required HLA allele will be able to respond to the therapy, which substantially limits its application [152]. In the case of CRC, numerous tumor-associated antigens have been identified and utilized in order to target MHC class I. Among these antigens are carcinoembryonic antigen (CEA), mucin-1, squamous cell carcinoma antigen recognized by T cells 3 (SART3), β-human chorionic gonadotropin (β-hCG), Survivin-2B, and p53 [153,154]. However, clinical trials have shown mixed results even though the overall trend was positive [155,156].

Viral vector vaccines: Due to the relatively low stimulating capacity of peptide antigen vaccines, several teams have tried to augment the generated immune response via the use of viral or bacterial vectors. In fact, a recombinant virus engineered to express tumor-associated antigens utilizes the inherent immunogenicity of viruses, as they tend to naturally infect antigen-presenting cells, particularly dendritic cells (DCs). Taking it a step forward, the co-introduction of tumor antigens along with co-stimulatory molecules in a viral vector seems to be an even more effective way to trigger an effective immune response [157]. Such an example is the CEA/TRICOM vaccine which utilizes CEA as the target protein and combines this with three co-stimulatory molecules (TRICOM) B7.1 (CD80), intercellular adhesion molecule 1 (ICAM-1), and lymphocyte function-associated antigen 3 (LFA-3) all packed within a viral vector [158]. Interestingly this type of vaccine has given some promising results as evidenced in a murine model of colon cancer [159].

Dendritic cells vaccines: While the effectiveness of previous categories relies upon the stimulation of the immune system in order to elucidate an anti-tumor immune response, the final category utilizes autologous DCs in order to orchestrate the anti-tumor response. This can be achieved because DCs have the potential to activate T cells through antigen presentation by MHC. One way to achieve this is via the generation of ex vivo DCs capable of activating anti-tumor CTLs through the isolation of CD14+ monocytes from peripheral blood. Following this, the next step is to differentiate and manipulate them into antigen-presenting mature monocyte-derived DCs (moDCs) which are then delivered back to the patient [160]. Despite the promising responses observed, a significant portion of patients have not experienced clinical benefits. It could be argued that moDCs may have an inherently suboptimal ability to provoke T-cell responses [161]. Unfortunately, ex vivo DC differentiation and manipulation is time-consuming and expensive, while also available in very few laboratories [70].

## 9. Emerging Biomarkers of Clinical Response

In current clinical practice, various biomarkers such as PD-L1 expression, tumor mutational burden (TMB), MSI, microbiome composition, hypoxia levels, IFN-γ levels, and extracellular matrix characteristics are utilized as prognostic indicators to predict the clinical response to immunotherapy among patients receiving ICIs [162]. Recent findings suggest that assessing the densities of T and B cells within CRC tumors offers higher accuracy compared to evaluating PD-L1 expression alone in predicting the efficacy of immunotherapy. This is because PD-L1 expression can vary heterogeneously within different regions of a tumor, while metastatic CRC lesions may exhibit varying immunoscores [163,164]. Despite significant advancements in identifying biomarkers for ICIs, Fucà et al. conducted a study evaluating the impact of metastatic site location on clinical response. Their findings revealed that the presence of peritoneal metastases and ascites correlated with poorer outcomes in a cohort of 502 patients with dMMR/MSI-H mCRC treated with ICIs. This association has been attributed to the presence of an immune-suppressive environment within serous cavities, potentially hindering the efficacy of immunotherapy [165]. POLE and POLD1 enzymes play pivotal roles in DNA replication, crucial for synthesizing and repairing DNA. The exonuclease domain (ED) of POLE and POLD1 has the ability to repair replicating errors of the DNA. Therefore, mutations located in the DNA binding or catalytic site of the ED of POLE or POLD1 can lead to proofreading defects. Consequently, increased TMB is closely related to an absent or impaired DNA repair system that results in an ultra-mutated phenotype of tumor cells. For this reason, the abundance of mutation-associated neoantigens identified in tumors with mutations in POLE or POLD1 makes them heavily immunogenic. This, in turn, allows them to be potentially sensitive to immune checkpoint inhibition [166]. Previous work on this topic has shown that pMMR/MSS tumors baring POLE- or POLD1-mutations are often diagnosed at earlier stages and exhibit favorable prognoses compared to cases with wild-type POLE or POLD1 [167]. POLE-mutated tumors are characterized by certain elements such as infiltration of CD8+ T cells at higher rates, greater expression of cytotoxic markers, and upregulation of immune checkpoints in contrast to POLE wild-type pMMR/MSS tumors. Additionally, they demonstrate a similar enrichment of CD8+ T cells as observed in tumors with dMMR/MSI-H phenotypes, suggesting a similar underlying biology between these two subgroups of CRC tumors [167].

## 10. Dynamics of Telomere Length

Chromosomal ends consist of special DNA structures named telomeres. These are created by the repetition of certain nucleotide sequences (5′-TTAGGG-3′) [168]. These sequences are non-coding, but along with other proteins (shelterins), protect from chromosomal damage, instability, and degradation [168]. Shelterin is a complex of proteins comprising six subunits—TRF1, TRF2, TIN2, TPP1, and POT1 [169]. Together, shelterins and telomeres create T-loops, keeping DNA repair mechanisms from identifying telomeres as double-stranded DNA breaks. However, during each mitotic cell division, DNA polymerases are unable to fully replicate the lagging strand of telomere DNA at the chromosome end, resulting in an annual telomere shortening rate of approximately 20–40 base pairs [170,171]. Additional factors (processing of telomeres during the cell cycle, reactive oxygen species) also worsen telomere shortening [172]. Combined, these events initiate a DNA damage response that creates two key situations: (1) introduces irreversible cell cycle arrest, and (2) initiates cellular senescence that is characterized by changes in chromatin, gene expression, organelles, and cell morphology [173]. Nevertheless, despite the theory of telomere attrition seeming logical, it is not able to explain senescence in non-proliferating, quiescent, or terminally differentiated cells, suggesting a more complicated pathway [174]. Telomerase, an enzyme with reverse-transcriptase action, can counteract this loss of telomeric length by synthesizing telomeric DNA elements using an RNA template [175]. Telomerase comprises a number of proteins such as telomerase reverse transcriptase (TERT) and telomerase RNA component (TERC), along with various proteins essential for DNA synthesis. Despite this, TERC is ubiquitously expressed in various human cells, and TERT is suppressed in order to prevent uncontrolled cell replication [176]. In addition, it is well established that due to its suppression, TERT acts as a rate-limiter of telomerase activity [177]. This is important because if TERT is upregulated, then TA will be increased, allowing cells to overcome replicative senescence. Doing so, telomeres become dysfunctional, which allows for continuous “breakage-fusion-bridge” cycles, chromosomal fusions, gene amplifications, derived chromosome imbalances, and the formation of complex non-reciprocal translocations to take place, a hallmark feature of adult solid tumors and genomic instability in general [178]. However, neoplastic cells may also achieve genomic instability through short telomeres. In fact, it is believed that at the early stages of neoplastic transformation, telomere shortening reaches a critical length, at which senescence would be triggered. However, oncogenic changes help the cell to bypass senescence and continue divisions until multiple critically shortened telomeres initiate crisis (a period of complete replicative senescence, chromosome end-to-end fusions, and extensive apoptosis). If the neoplastic cell has acquired certain oncogenic modifications, it will overcome the crisis and continue to replicate. Eventually, in many cases, hTERT will be upregulated and TA will be increased, leading to longer TL [179]. This fulfills one of the ten hallmarks of carcinogenesis (as described by the Hanahan–Weinberg model for successful oncogenesis [180]), which is the initiation of replicative immortality. In mutated cells, TERT is upregulated owing to the carcinogenic properties of transcription factors Myc, nuclear factor kappa B (NF-kB), and b catenin [181]. It has to be noted though that apart from initiating replicative immortality, telomerase seems to play a crucial role in almost all the hallmarks of cancer, ranging from cell growth and proliferation through to the activation of EGFR signaling [182], resistance to apoptosis through activation of NF-kB [183], and the promotion of invasion and metastasis through induction of the epithelial–mesenchymal transition via the Wnt/b-catenin pathway that upregulates Snail-1 and vimentin [184], up to direct and indirect (through Myc) modification of energy metabolism [185]. It has been shown that homeostasis of the telomeric ends can be regulated by protein parts of the shelterin complex [186,187]. Two different key regulators of TA are the actual structure of telomeres and the cell cycle-dependent creation of active telomerase. Both of them seem to restrict TA at chromosome ends during the S phase of each cell cycle [188]. However, it is rather interesting that during each cell cycle, TA seems to be concentrated primarily to these telomeric ends whose length reaches a critically short length (telomeric prioritization) [22,189]. The level of TA and the corresponding TL are the two major elements affecting telomere homeostasis. Throughout the cell cycle, telomeres fluctuate between different states (stationary/nonextendible, elongetable, and elongating) [188]. Moreover, recent data indicate that telomeres in mammals have the ability of transcription into telomeric repeat-containing RNA (TERRA) molecules, representing a novel type of mammalian RNAs. This comes in contrast to the notion that telomeres are non-coding regions of the DNA [190]. In fact, telomere transcription seems to be mediated from within the telomere region of CpG islands with promoter activity (identified within subtelomeric regions). These promoters have been found to be able to initiate transcription of TERRAs, containing UUAGGG repeats [191]. Despite a lack of universal consensus regarding the main function of TERRAs, this seems to be the formation of telomeric heterochromatin, the protection of telomeres, and the negative regulation of telomerase [192].

## 11. Telomere Length and CRC

Cancer cells can overcome cell cycle arrest and activate telomerase, allowing them to proliferate and acquire mutations. In cancer research, significant attention has been given to understanding the regulation of the human telomerase reverse transcriptase (hTERT), which plays a crucial role in cancer. Various mechanisms have been identified to alter hTERT gene expression, including methylation of its promoter, mutational changes within the promoter, amplifications, and structural variations [193]. Recent studies on CRC have revealed that TL from malignant cells tends to be shorter than in neighboring “healthy” mucosa [194]. Additionally, the clinical stage of the malignancy seems to be correlated with TL. In fact, cancers at lower stages tend to have shorter telomeres in contrast to advanced and metastatic cancers [194]. The correlation between TL and cancer has been widely demonstrated, with more than 6000 peer-reviewed scientific and clinical publications in this specific field. In a recent study, Nikolouzakis et al. showed that TL in PBLs from patients with mCRC tends to decrease with age (especially for patients over 75 years old) when compared to healthy individuals of the same age, while chemotherapy seemed to also have a negative impact on the mean TL of mCRC patients [29]. In this study, metaphase Q-FISH technology was used for the determination of TL in PBL samples. This technique is the most accurate and precise compared to others since it measures each telomere end in each chromosome in each cell, avoiding the false measurement of the interstitial telomere regions of the chromosomes, and allows the recognition of telomere-free ends which lead to chromosomal instability and are linked with age-related diseases such as colon CRC [195,196]. Some researchers, such as Luu et al. [197] and Peacock et al. [198], have reported that the risk of developing CRC seems to be higher in people with longer telomeres. An explanation they provide dictates that there is an increased risk of mutational accumulation with carcinogenic potential as a result of longer telomeres. Nevertheless, this theory seems to be wrong, as a meta-analysis failed to identify any significant association between TL and the risk for CRC development [199]. Regarding TL and tumor location, while earlier findings suggested that rectal cancers had a tendency to bear longer telomeres [200], a formal correlation between TL and tumor location is still debated. Furthermore, TL has emerged as a potential predictive biomarker for the clinical outcome of anti-EGFR monoclonal antibody therapy in patients with KRAS wild-type metastatic CRC. Therefore, investigations are underway to determine whether mutations in the KRAS oncogene impact telomere deregulation [21,24,27,29,133].

## 12. Telomerase Activity and CRC

TA in CRC has been detected in a significant percentage of tumor samples, ranging from 80% to 100% [201], highlighting its role as a pivotal step in CRC carcinogenesis [202]. In contrast, TA has been found in normal mucosa samples at a lower frequency, ranging from 9% to 53% [22,203]. Across numerous studies, neoplastic mucosa consistently exhibited higher TA compared to corresponding healthy mucosa. However, Nikolouzakis et al., studying TA in PBLs from 28 patients with mCRC, described that TA presented a significant trend of increase (*p* = 0.07) in patients with progressive disease at the middle of their treatment when compared with that before the initiation [27]. Researchers have identified TA as an independent prognostic factor for DFS, recurrence, and OS in CRC patients, with high levels of TERT and/or TA typically associated with poorer prognosis [204]. Studying TA indirectly can be achieved by assessing hTERT expression, as its acquisition appears essential for TA in most human tumors [205]. There is a strong correlation between hTERT expression and TA, and recent studies have linked hTERT promoter hypermethylation to CRC and other cancer types [206]. Additionally, elevated levels of hTERT have been linked to OS in CRC patients [207]. In fact, the identification of hTERT transcripts in the plasma of CRC patients may serve as a useful tool for disease monitoring, as mRNA levels correlate with those found in tumors [208]. The prognostic and predictive significance of cell-free circulating hTERT has been evaluated in CRC patients in detail [207,209,210]. Bertorelle et al. showed that stage II CRC patients with lower levels of hTERT had better OS, while its presence at higher levels was correlated with a poor response to chemotherapy (as an independent prognostic factor) and with a higher risk of disease recurrence and death [207]. However, owing to the fact that the extracellular compartment contains RNases, the estimation of circulating hTERT transcripts is unreliable due to mRNA digestion [211]. For this purpose, exosomes containing hTERT can be evaluated. Exosomes are small (30–150 nm) particles arising from the endosomal membrane compartment. They contain miRNA, mRNA, proteins, DNA, and lipids. It has been shown that a variety of both normal and neoplastic cells can secrete them (among which are CRC cells) into the microenvironment from where they enter the bloodstream. Exosomes offer the advantage that their mRNA content is not exposed to the activity of extracellular RNases, allowing for reliable estimation [210]. In summary, ample evidence supports telomerase as a useful marker for monitoring and predicting outcomes in CRC [207].

## 13. Therapeutic Implications of Telomerase Activity Inhibition

Anti-telomerase cancer therapy aims to inhibit or suppress TA in cancer cells through various inhibitors targeting different pathways. Currently, the primary telomerase inhibitor in clinical trials is GRN163L, an antisense oligodeoxynucleotide that complements the template region of human telomerase RNA (hTR) [212]. By blocking telomerase’s catalytic activity, GRN163L induces progressive telomere shortening, leading to senescence and apoptosis. Preclinical studies in vitro and in vivo have paved the way for phase II human clinical trials [212,213]. Cancer immunotherapy offers another avenue for anti-telomerase inhibition by targeting telomerase-positive malignant cells. hTERT serves as a tumor-associated antigen, generating peptides that, when presented with class I major histocompatibility molecules, can stimulate cytotoxic T-lymphocytes to attack cancer cells. Several phase I and II clinical trials utilizing TERT-directed vaccines have shown promising results [213,214]. The EGFR pathway plays a crucial role in regulating the telomerase complex [215]. Antisense oligonucleotides targeting EGFR have demonstrated significant inhibition of TA, along with TL reduction in various cell lines [216]. Studies have revealed the coexistence of EGFR expression and TA in human uterine cervix epithelial cells [217]. Additionally, downstream targets of EGFR signaling, such as the hypoxia-inducible factor 1-alpha (HIF1-α) and the PI3K/AKT pathway, contribute to the transcriptional regulation of hTERT, further linking EGFR activation to telomerase regulation [218]. TL has emerged as a potential predictive biomarker of clinical outcome, particularly progression-free survival (PFS), in response to anti-EGFR therapy in patients with KRas wild-type mCRC [219]. Augustine et al. demonstrated in vitro that shorter TL correlated with restricted growth in response to cetuximab (an anti-EGFR antibody), while patients with longer TL exhibited a longer PFS when treated with EGFR inhibitors (cetuximab/panitumumab) [220]. Integrating TL into biomarker screening alongside Ras mutations could refine patient selection for anti-EGFR therapy, optimizing treatment efficacy [219]. The extensive literature linking the EGFR pathway with the TL/telomerase complex underscores the potential utility of TL as a predictive marker in anti-EGFR therapy [218].

## 14. Potential Interplay between Telomere Length and Immune Therapies

Various environmental drivers or chronic diseases have the potential to accelerate the physiological aging process related to telomeres in leukocytes [221]. Furthermore, maintaining a consistently healthy lifestyle has been proposed as a strategy to alleviate the impact of hazardous effects on TL [171,222]. Additionally, the proinflammatory milieu of tumors and their microenvironment may add to the overall negative impact of telomere shortening. Evidence suggests that telomere shortening can be triggered by age-independent immune senescence that is caused by tumor-associated inflammation [223]. It is well established that mature cells of myeloid lineage do not undergo division as compared to mature lymphocytes. Therefore, it is expected that the TL measured in leukocytes reflects cumulative or average lengths over cells with potentially diverse replications. Moreover, TL naive T cells are longer than that of memory T cells [224]. The same stands for CD28+ T cells as compared to more differentiated lines such as CD28− or CD8+ T cells [225,226]. Cross-sectional analyses demonstrate progressive shortening of TL IN with advancing age in leukocytes or peripheral blood mononuclear cells (PBMCs), as well as in isolated granulocytes and lymphocytes (CD4 and CD8 T cells, and B cells) [227]. According to the above observations, it can be stated that telomere attrition in blood leukocytes of both lineages (myeloid and lymphoid) is a fact, with TL being dependent upon age and differentiation. Some individuals exhibit a suboptimal response to vaccines, possibly due to various factors. Dysfunctional telomeres may contribute to this phenomenon. As observed in chronic viral infections, repetitive stimulation leads to extensive proliferation and the shortening of telomeres in antigen-specific T cells, ultimately leading to exhaustion [228]. According to Rolles et al., patients with a longer TL had better OS as compared to those with shorter telomeres, regardless of the type of cancer. Moreover, it has to be noted that TL had no effect on patients’ response to ICI therapy. The authors assumed that the observed difference in OS can be explained by the fact that shorter telomeres may be associated with increased non-tumor-related mortality.

## 15. Conclusions

As the diagnosis of CRC increasingly affects a younger population and the prevalence of advanced stages of the disease rises, the imperative to expand CRC treatment modalities becomes ever more pressing. Substantial progress has been achieved in elucidating the immune system’s pivotal role in cancer pathogenesis, including CRC. This analysis has revealed two noteworthy trends that are presently shaping and will persistently influence cancer therapy in the foreseeable future: the employment of immune cell markers for predicting cancer outcomes and the targeting of diverse aspects of the immune system to induce an anti-tumor immune response. Immunotherapy for CRC has mainly been integrated as an adjuvant therapy alongside established first-line approaches, primarily directed at patients with dMMR/MSI-H and/or mCRC. Numerous investigations are ongoing to broaden the applicability of CRC immunotherapy, exploring additional biomarkers or microenvironments that modulate the anti-tumor immune response and evaluating immunotherapy, particularly ICIs, as both a primary and standalone mode of CRC treatment. Simultaneously, TL and TA have already demonstrated their clinical relevance as prognostic and/or predictive biomarkers of response in CRC therapy, while therapeutic strategies targeting hTERT are also under scrutiny. However, TL and TA also serve as crucial indicators of immune status. Consequently, interventions aimed at enhancing TL/TA hold promise as adjunctive approaches to enhance the response to anti-cancer immune therapy.

Pearls:

(1) In cases of early-stage CRC, the dMMR/MSI-H profile is more common (10–18%) than that of mCRC (about 3–5%).
(2) Activation and inhibitory signals of T cells are mediated through CTLA-4 [229].
(3) A suppression of immune responses is exhibited by the interplay of PD-1 and PD-L1/PD-L2, inducing a minimized function of T effector cells over immune responses.
(4) PD-L1 is highly expressed in inflamed cells and PD-L2 is expressed only in antigen-presenting cells [230].

## Figures and Tables

**Table 1:** Presentation of the different categories and types of therapeutic applications for colorectal cancer. Immune checkpoint inhibitors (ICIs), adoptive cell therapy (ACT), chimeric antigen receptor (CAR) T-cell, tumor-infiltrating lymphocyte (TIL).

Colorectal Cancer Immunotherapy				
Category	ICIs		ACT	Anti-Tumor Vaccines
Type	Anti PDL1Abs	Atezolimumab	CAR-T	Whole tumor
		Avelumab		
		Durvalumab		
	Anti PD-1 Abs	Pembrolizumab	TILs	Peptide antigen
		Nivolumab		
		Cemiplimab		
	Anti-CTLA-4 Abs	Ipilimumab		Viral vector
				Dendritic cells

**Table 2:** Presentation of the main categories of immune-mediated anti-tumor vaccine platforms along with their advantages and disadvantages.

Anti-Tumor Vaccines				
Type	Sample	Advantages	Disadvantages	Conclusion
Whole tumor	Cancer tissue (Autologous)	Patient-specific	Cross reaction with normal cells	No significant benefits in OS/DFS
Peptide antigen	Tumor-specific peptides (Heterologous)	Less cross reactions with normal cells	Low immunogenicity HLA specific	Positive results
Viral vector	Recombined Tumor-specific peptides (Heterologous) with viral/bacterial vectors	Less cross reactions with normal cells Increased immunogenicity		Positive results
Dendritic cells	Dendritic cells (Autologous)	High specificity	Time-consuming Expensive Low availability	Mixed results
